# Defining a Link between Perceptual Learning and Attention

**DOI:** 10.1371/journal.pbio.0060221

**Published:** 2008-08-26

**Authors:** Yuko Yotsumoto, Takeo Watanabe

## Abstract

Takeo Watanabe and Yuko Yotsumoto explore the implications of a new study that shows that for perceptual learning of visual features involving multiple stimuli to occur, the brain needs to temporally "tag" the features, a learning process that requires paying attention.

Experts show amazingly high perceptual skills. Experienced jewelers routinely classify diamonds that appear very similar to the uninitiated into different grades with high precision. Within a few seconds, airport baggage security officers can detect forbidden inconspicuous materials through x-ray images. Such feats are possible because the experts' “eyes” are trained through practice and experience. Long after most aspects of brain development have ceased, repeated exposures or trainings improve our perceptual/sensory abilities, and cause neural reorganizations in the brain. Such experience-induced improvement, called perceptual learning [1], and the accompanying neural changes, called neural plasticity [2–6], underlie not only our ability to master a trade but operate at a more fundamental level to help us make sense of the world.

We are constantly exposed to an overwhelming amount of sensory signals, most of which are not noteworthy. To function normally in the world, we must react quickly and precisely to the important signals, while ignoring or discounting the less important, just as organisms must do in the natural environment to survive. By directing attention only to important signals or being repeatedly exposed to signals in an important context, our sensory systems learn to process important signals more efficiently than the less-important signals. Reflecting this fundamental role of perceptual learning, studies have been conducted to examine mechanisms of perceptual learning and neural plasticity with various kinds of tasks and stimuli by using behavioral measurements [2,3] and neurophysiological [7,8] and brain imaging techniques [9,10]. Perceptual learning and neural plasticity have also been studied in all the sensory modalities including vision, hearing [11], and touch perception [12].

## Models of Perceptual Learning

**Figure 1:**
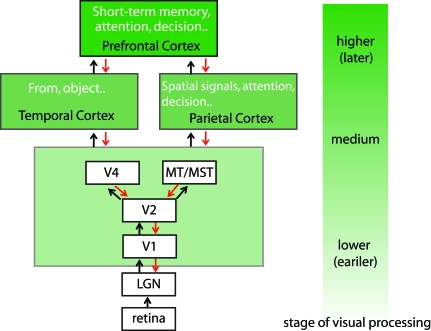
Visual Information Processing Visual processing is conducted at many different levels in a hierarchical manner. Light strikes the retina and is converted into electrical signals. They are then sent to the lateral geniculate nucleus (LGN) in the subcortex and further projected onto the primary visual cortex (V1), where primitive features including orientation and spatial frequency (coarseness) in the signals are processed highly locally. The signals are further sent to higher cortical areas. At a higher level, more abstract and complex features tend to be processed more globally. Spatial/motion visual signals are predominantly processed in the parietal areas and shape/object signals are predominantly processed in the temporal areas. Then the signals are used for decision making in parietal and prefrontal areas. However, the hierarchical processing from lower to higher areas (black arrows) is just one aspect of visual information processing. First, signals also flow from higher to lower areas (red arrows). For example, attention that may originate at prefrontal and/or parietal areas exerts top-down signals to low-level visual areas. Second, within the same visual area spatially remote regions can interact with each other, following initial local processing.

**Figure 2:**
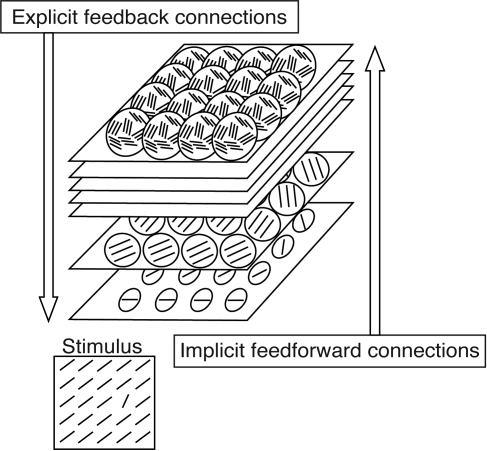
Schematic Representation of RHT [20]. Easily discriminable signals are processed in higher visual stages. When the higher stages fail to process the signals due to finer differences, the signals are processed in lower stages. Learning is driven by attention.

To explore the mechanism of perceptual learning, here we focus on visual perceptual learning. Visual processing consists of many different stages leading from eyes to cortical areas for cognitive processes such as decision making (Figure 1). It is unlikely that all types of visual perceptual learning involve the same cortical stage(s). The stage(s) in which one type of visual perceptual learning occurs may depend on many factors, including the learned visual feature such as orientation and contrast, the type of tasks such as a detection task or a discrimination task, and exposure to a feature without a task. For instance, some types of visual perceptual learning may only involve lower stages of visual processing, such as V1, while other types of visual perceptual learning may involve multiple stages of visual processing. Models of different mechanisms are proposed depending on the stage(s) visual perceptual learning involves.

(A) *Early stage, local network model*: Adini et al. [13] and Tsodyks, et al. [14] have proposed the model based on perceptual learning of contrast discrimination (indicating whether two contrast values are the same or different). In their model, repeated presentation of a stimulus (a line presented in the center) together with its surrounding stimulus (lines presented around the central line) leads to a change in the visual cortex as a result of interactions between signals from the central and surrounding stimuli. That is, for an observer to learn a stimulus in one location (the central stimulus), the context in which the stimulus is presented (surrounding stimuli) plays an important role. The interactions can occur within the same cortex and therefore we do not have to assume interactions between cortical areas at different levels in the visual processing hierarchy. In this model, the neural reorganization due to perceptual learning can occur in a low-level cortex, including the primary visual cortex (V1), which is the first visual cortex onto which visual signals are projected. This model indicates the mechanism of a type of perceptual learning that can involve only one level of visual processing and suggests that perceptual learning does not necessarily require lower-to-higher or higher-to-lower connections between different cortical areas at different stages of visual processing.

(B) *Mid-level stage, reweighting*: In the model by Dosher and Lu [15–17], learning occurs by changing the strength (reweighting) of neural connections between the early visual stage, such as V1, in which highly local processing occurs, and a decision unit. The changes occur in the neural connections specifically for a given task. In this sense, it is possible that different stages between the earliest visual stage and the decision unit are involved.

(C) *Higher-to-lower stages*: Reverse hierarchy theory (RHT) indicates that learning is an attention guided process [18–20] (Figure 2). According to this model, visual learning begins at high-level visual areas that may be able to deal with a task requiring discriminating signals with large differences (Figure 1). When a task requires discrimination of signals with smaller differences, the site of learning proceeds to lower visual areas where signals with smaller differences can be discriminated. RHT indicates that learning is driven by attention that selects neuronal population suitable for the levels of the signal.

The three models fit well with different types of perceptual learning that take place in different stages of the visual processing stream (Figure 1).

## Perceptual Learning of Multiple Values of Sensory Features

While the aforementioned models deal with perceptual learning of a single feature value, in reality, we are exposed to wealth of different stimuli and we switch our attention from one stimulus to another. Thus, we are constantly exposed to multiple values of sensory features (a range of different orientations or different values of brightness). How can we learn these different values of a feature? In a new study in *PLoS Biology*, Zhang et al. conducted a series of experiments to address this issue [21]. They used contrast or orientation discrimination tasks [22], in which multiple values of a feature (e.g., a grating pattern with contrast of 0.2, and a grating pattern with contrast of 0.3) were learned. Their results show that, to learn multiple values of a feature, different stimuli with different values of a feature need to be presented in a temporal pattern. For instance, learning occurred when different values of a feature were presented with a fixed inter-trial interval, whereas learning failed when they were presented with random intervals. The researchers also demonstrated that if a specific value of a feature was followed, or tagged, with a specific letter such as an “A” or “B” in every trial, learning of multiple values of the feature occurs even if they are presented in a random order.

Based on these findings, Zhang et al. have proposed the “stimulus tagging model” for perceptual learning. This model indicates that to learn multiple stimuli, the brain needs to conceptually “tag” each stimulus that differs in the feature value. A presentation of feature values in a structured way over time or a presentation of the same label paired with a specific value of a stimulus feature is regarded as tagging. For example, the letter “A” is paired with one feature value and the letter “B” with another feature value. Thus, tagging can be conceptual. Such tagging guides attention directly to the appropriate perceptual template.

How is the stimulus tagging model applied to real life situations? Imagine that a young animal is learning to visually discriminate multiple kinds of fruits. While all the fruits look alike, only one of them is edible. To learn to visually discriminate them, the animal could “tag” based on the different tastes of each fruit.

In the stimulus tagging model, the tagging can be conceptual or semantic. Thus, the “stimulus tagging” is likely to involve higher stages of processing, such as the prefrontal cortex, while a given visual task such as discrimination of multiple feature values involves lower stages of visual processing, such as visual cortex. Therefore, the effect of “tagging” on perceptual learning indicates top-down (higher-to-lower levels) modulation. In that sense, the stimulus tagging model is in accord with the RHT model in which attention drives learning by engaging appropriate population of neurons. On the other hand, while neither the local network model nor reweighting model assumes involvement of top-down processing as necessary for occurrence of perceptual learning, they do not deny the involvement of top-down processing.

## Attention and Perceptual Learning

Note that “attention” in the tagging model involves assignment of a conceptual label. This makes one wonder what types of attention are involved in the tagging process. Posner and colleagues [23-25] defined attention as assemblies of three different subsystems: alerting, orienting, and executive attention. Alerting is characterized as achieving and maintaining an alert state. Orienting is characterized as selection of information from sensory input by directing attention to a cued spatial location. Executive control of attention is characterized as resolving conflict between signals that do not induce the same response. For example, the executive control selects signals relevant to a given task while filtering out task-irrelevant signals that conflict with the relevant signals. While these subsystems often operate simultaneously with some interactions, basically they are orthogonally constructed systems that can operate independently [24].

In the study by Zhang et al. [21], successful perceptual learning of multiple feature values occurs when the “tagging” successfully differentiated the feature values. For example, both presenting a letter “A” while discriminating contrasts around 0.2, and presenting a letter “B” while discriminating contrasts around 0.3 allowed the participants to learn to discriminate both contrasts around 0.2 and contrasts around 0.3. Since tagging was conceptual or semantic without needing to involve spatial information or conflicting signals, the type of attention involved in tagging is not likely to be either orienting that works for spatial information or executive control that deals with conflicting signals. The remaining type of attention is alertness. How does alertness play a role in perceptual learning with tagging?

Seitz and Watanabe have suggested that when a stimulus is presented concurrently with internal alertness elevation, perceptual learning of the stimulus occurs [26]. Perceptual learning with stimulus tagging might occur within this framework. When a previously presented temporal pattern or label is presented and successfully distinguished from other patterns or labels, the participant's alertness level might be elevated, and features presented concurrently with the pattern or label may be learned.

Clarifying the role of attention in perceptual learning provides clues to elucidate the underlying neural mechanisms of perceptual learning. Seitz and Watanabe [26] pointed out a potential link between perceptual learning and the alerting system by noting that the alerting system is associated with the right frontal regions and the right parietal regions [27-32]. For instance, lesions in the right frontal regions or the right parietal regions cause deficits in attention most related to alertness. In these areas, the norepinephrine system is thought to be involved in the activations [33,34]. On the other hand, neural activity in the locus coeruleus (LC), from where norepinephrine arises, is correlated with behavioral improvements on visual discrimination tasks [35]. A study has indicated that neural activities in the LC closely correlated with fluctuations in behavioral performance of visual discrimination task [36]. How perceptual learning and alerting attention are related, particularly in terms of the norepinephrine system, would be a highly interesting subject of future investigations.

Another potential future extension of the stimulus tagging model would be whether it can be generalized to other modalities. Although the tagging model is based on results of visual perceptual learning, it would be highly interesting to examine how well the tagging model fits with learning models associated with other sensory modalities, including audition and tactics, that have a hierarchical signal processing structure and top-down modulation just like the visual system.

The study by Zhang et al. [21] has not only provided new behavioral findings about an important nature of perceptual learning, but has also built an interesting model that strengthens a link between perceptual learning and attention that can now be explored in future behavioral, neurophysiological, and neurobiological studies on perceptual learning.
